# Microfluidic Synthesis of Rigid Nanovesicles for Hydrophilic Reagents Delivery**

**DOI:** 10.1002/anie.201500096

**Published:** 2015-02-20

**Authors:** Lu Zhang, Qiang Feng, Jiuling Wang, Jiashu Sun, Xinghua Shi, Xingyu Jiang

**Affiliations:** Beijing Engineering Research Center for BioNanotechnology & CAS Key Laboratory for Biological Effects of Nanomaterials and Nanosafety, National Center for NanoScience and TechnologyNo.11 ZhongGuanCun BeiYiTiao, Beijing, 100190 (P. R. China); State Key Laboratory of Nonlinear Mechanics, Institute of Mechanics, Chinese Academy of SciencesNo.15 Beisihuanxi Road, Beijing, 100190 (P. R. China)

**Keywords:** drug delivery, hydrophilic reagents, microfluidics, nanoparticles, vesicles

## Abstract

We present a hollow-structured rigid nanovesicle (RNV) fabricated by a multi-stage microfluidic chip in one step, to effectively entrap various hydrophilic reagents inside, without complicated synthesis, extensive use of emulsifiers and stabilizers, and laborious purification procedures. The RNV contains a hollow water core, a rigid poly (lactic-co-glycolic acid) (PLGA) shell, and an outermost lipid layer. The formation mechanism of the RNV is investigated by dissipative particle dynamics (DPD) simulations. The entrapment efficiency of hydrophilic reagents such as calcein, rhodamine B and siRNA inside the hollow water core of RNV is ≈90 %. In comparison with the combination of free Dox and siRNA, RNV that co-encapsulate siRNA and doxorubicin (Dox) reveals a significantly enhanced anti-tumor effect for a multi-drug resistant tumor model.

Rigid nanoparticles are effective for delivery of therapeutic agents. We have shown that rigid PLGA-lipid nanoparticles (PLGA=poly (lactic-*co*-glycolic acid)) loaded with hydrophobic drugs display an enhanced cellular uptake and cancer treatment compared to flexible PLGA-lipid nanoparticles of similar properties.[1] In addition to hydrophobic therapeutic agents, hydrophilic reagents, such as small interfering RNA (siRNA), plasmids, protein, some anticancer drugs, and some small molecular fluorescent probes, hold great promise in molecular biology and pharmaceutical sciences. Compared to hydrophobic drugs, nanocarriers for hydrophilic chemicals are generally required because: 1) some hydrophilic reagents such as nucleic acids are reluctant to be uptaken by cells and unstable without encapsulation;[2] 2) some hydrophilic therapeutic agents show rapid metabolism and thus very short circulation half-life.[3] Despite of an explosive development of nanocarriers including liposomes,[4] block copolymers,[5] and hybrid nanoparticles[6] for hydrophobic drugs delivery, several issues arise in adopting nanocarriers for encapsulation of hydrophilic reagents (e.g., the relatively low entrapment efficiency of drugs,[7] low drug recovery rate,[8] limited applications of charge-based delivery systems,[9] and difficulty in synthesis of nanocarriers[8b,^10^]). To address the above challenges, a versatile, stable, and easy-to-fabricate nanocarrier for delivering hydrophilic chemicals is in high demand.[11]

Hybrid core–shell nanoparticles are becoming a new class of drug nanocarriers, which comprise a polymeric core, for example, PLGA, to carry hydrophobic drugs and reagents,[12] and an outer lipid shell to prolong the circulation half-life.[13] In contrast to bulk approaches usually involving complicated procedures and limited experimental control,[12a, 14] microfluidic platforms have been employed for controlled generation of hybrid nanoparticles in a rapid and straightforward manner.[15] However, an efficient encapsulation of hydrophilic drugs will necessitate a hollow structure. To synthesize the hollow core–shell nanoparticles, the formation of reverse micelles is always involved, which can only be stable for a very short time without the protection from emulsifiers and stabilizers. In bulk fabrication of reverse micelles by the double emulsion method,[14c] the extensive use of emulsifiers and stabilizers may lead to the increased toxicity, and the following purification process is complicated. The microfluidic approach may facilitate the generation of hollow core–shell nanoparticles due to the precise fluids control and rapid mixing in small scales,[16] but has not been realized yet.

Here we report on a multi-stage microfluidic chip to manufacture water core/PLGA shell/lipid layer rigid nanovesicles (RNVs) to entrap varying hydrophilic reagents into the water core regardless of their properties such as molecular weight, surface charge, and so forth. We refer to this nanocarrier as rigid nanovesicle because compared to lipid/cell membrane-like vesicles with Young’s modulus of ≈1 MPa, the PLGA shell in RNV results in a much stiffer structure with Young’s modulus of ≈1 GPa.[4a] The microfluidic chip consists of three stages: 1) The first stage has three inlets and a straight channel (two side inlets for introducing PLGA and 1,2-dioleoyl-3-trimethylammonium propane (DOTAP) in *N*,*N*-dimethylformamide (DMF), and one center inlet for hydrophilic reagents such as siRNA, calcein and rhodamine B in water). 2) The second stage has two side inlets for water sheaths and one straight channel. 3) The third stage has one center inlet for DPPC, DSPE-PEG, and cholesterol in ethanol and a spiral channel (Scheme 1 and Figure S1 in the Supporting Information). In this work, the flow rate ratio (FR) of side inlets to middle inlet at different stages is optimized for synthesis of RNV, and the production rate of RNV is 114 µg min^−1^ by the microfluidic chip (Supporting Information). The transmission electron microscopy (TEM) image of an assembled RNV collected from the outlet shows a hollow core–shell structure with a bright water core and an intact PLGA shell (Figure 1 A). We use the positively charged DOTAP to form the reverse micelle as well as the inner surface of PLGA shell because DOTAP can interact with the negatively charged siRNA, thus ensuring an efficient encapsulation of siRNA inside the water core. Meanwhile, the lipids (DPPC, DSPE-PEG, and cholesterol) are assembled onto the outer surface of PLGA shell in the third stage of microfluidic chip, in order to achieve a long-term stabilization and a long circulation time.

**Figure 1:**
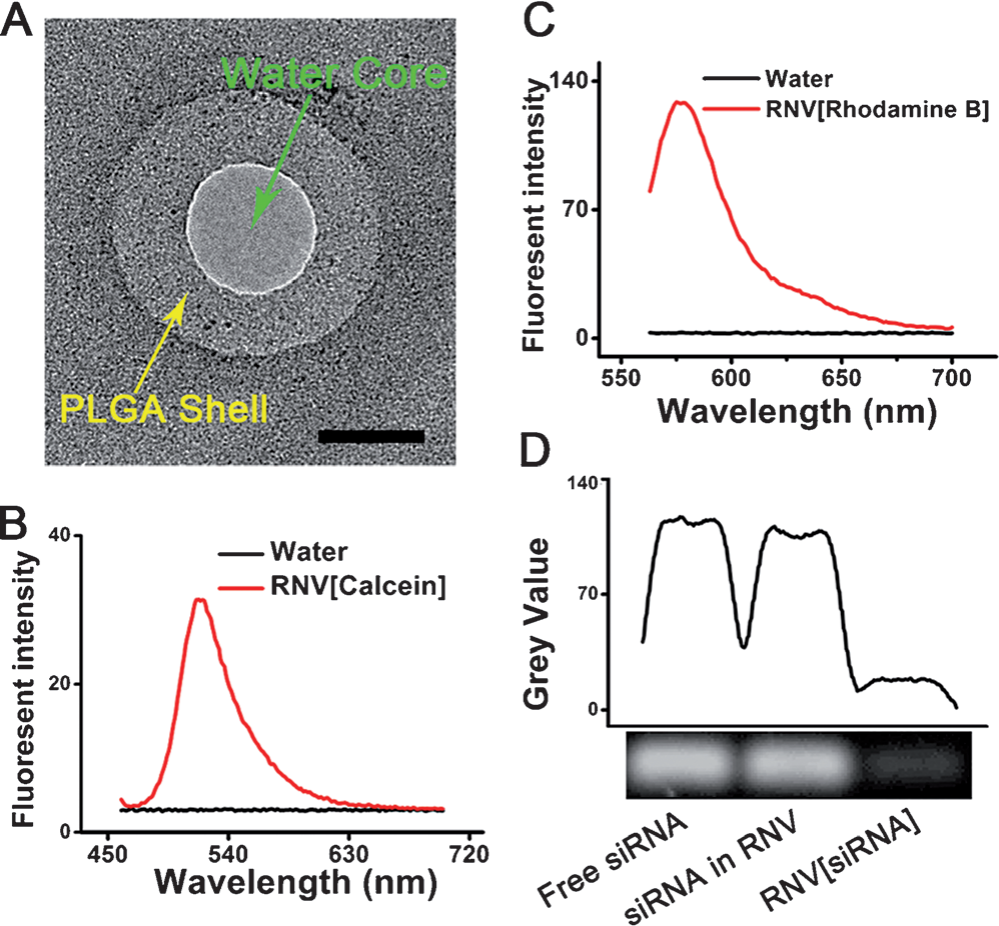
Characterization of the RNV encapsulating calcein, rhodamine B and siRNA. A) TEM image of a hollow core–shell RNV. Scale bar, 50 nm. B) Emission spectrum of RNV loaded with calcein excited at 455 nm. C) Emission spectrum of RNV loaded with rhodamine B excited at 556 nm. D) Gel retardation graph of free siRNA, lysed RNV[siRNA], and RNV[siRNA]. Lysed RNV refers to the RNV destroyed by three cycles of freeze–thaw and sonication to release the siRNA inside the water core.

**Scheme 1:**
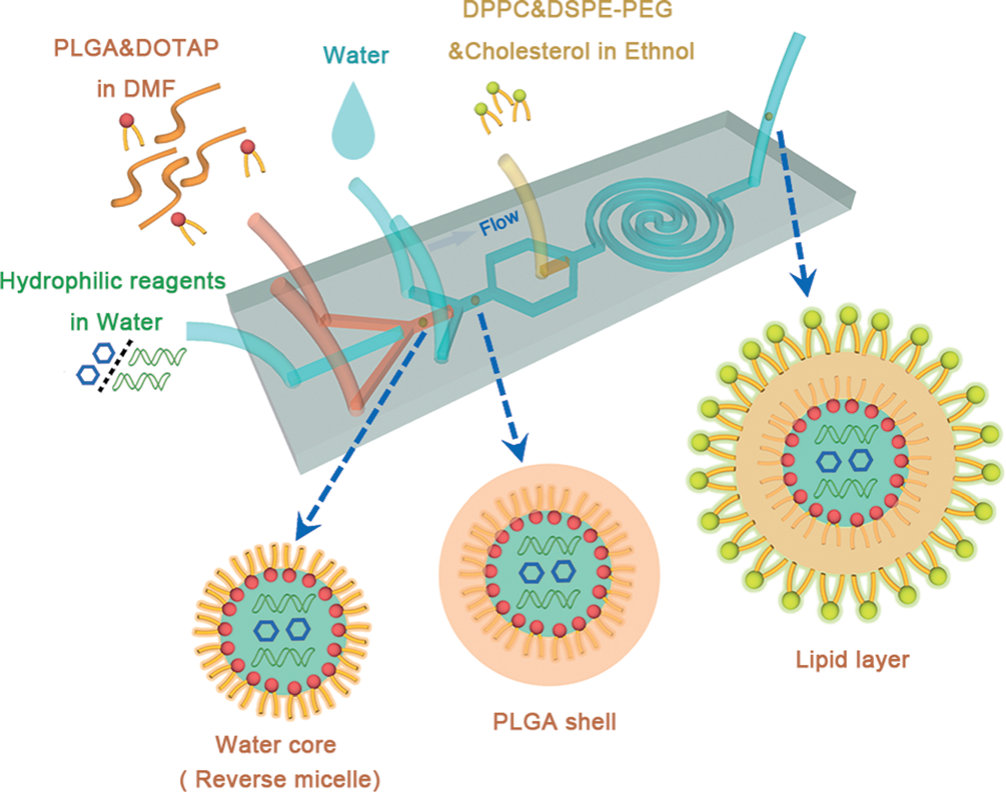
Schematic of the water core/PLGA shell/lipid layer rigid nanovesicle (RNV) assembled by the three-stage microfluidic chip in one-step.

We first encapsulate hydrophilic reagents, such as calcein, rhodamine B and siRNA, into the water core of RNV by microfluidic chips, and characterize the entrapment efficiency and the size of RNV. The entrapment of calcein (or rhodamine B) into the RNV and the entrapment efficiency are investigated using the spectrofluorophotometer and the microplate reader (Figure 1 B,C and Figures S2 and S3). To validate the encapsulation of siRNA into the RNV and quantify the entrapment efficiency of siRNA, we compare the gel retardation assays of free siRNA, lysed RNV loaded with siRNA (RNV is lysed by three cycles of freeze–thaw and sonication), and untreated RNV loaded with siRNA (RNV[siRNA], Supporting Information). The amount of siRNA released from the lysed RNV[siRNA] (the middle band) is almost the same as that of free siRNA control (the left band), while free siRNA signal (the right band) in the untreated RNV[siRNA] solution is barely observed, indicating the high siRNA recovery rate (≈98 %) and loading efficiency in RNV (Figure 1 D).

The measured hydrodynamic diameter of the RNV encapsulating different hydrophilic reagents is around 140 nm, and the polydispersity index (PDI) is smaller than 0.25 by dynamic light scattering (DLS) (Table 1). RNV loaded with different hydrophilic reagents shows marginal differences in size and PDI. The surface charges of the RNV encapsulating different reagents can be influenced by the contents. Briefly, the positively charged DOTAP may diffuse to the outer lipid layer, resulting in the positive surface charge of RNV when encapsulating calcein and rhodamine B. However, for fabrication of RNV encapsulating siRNA, the negatively charged siRNA will interact with the DOTAP, thus limiting the diffusion of DOTAP and resulting in the negative surface charge of RNV (Supporting Information). The entrapment efficiencies of three hydrophilic reagents into the RNV are almost 90 % (Table 1), approximately 1.5 times higher than bulk methods.[17] To investigate the degradation kinetics, we monitor the release curve of Dox encapsulated by the PLGA shell of RNV, which directly indicates the degradation of PLGA shell. The degradation of PLGA shell would ultimately result in the release of siRNA inside the water core of RNV (Figure S5). For the degradation of RNV, the PLGA shell plays a key role which forms a barrier to protect siRNA and holds the whole structure, while the lipids can further sustain the degradation as a result of the PEG chain.[8b]

**Table 1:** Size (*d*), zeta potential (*ξ*), polydispersity index (PDI) and entrapment efficiency (EE) of RNVs loaded with different reagents.

RNV/[reagent]	Z-AVE, *d* [nm]^[a]^	*ξ* [mV]^[b]^	PDI	EE [%]
RNV[rhodamine]	130.6±14.10	14.1±1.76	0.221±0.027	89.68±1.47
RNV[calcein]	149.8±2.815	11.2±0.35	0.132±0.018	90.66±0.27
RNV[Dox/siMDR1]	136.4±3.814	−14.2±3.25	0.222±0.017	91.23±4.51

[a] Z-AVE=mean particle size. [b] Details can be found in the Supporting Information.

To investigate the intermediates involved in the formation process of RNV within the microfluidic channel that are difficult to capture and characterize experimentally, we carried out dissipative particle dynamics (DPD) simulations, a tool to study dynamical behavior of vesicles and polymers on the molecular scale. In the first stage, we find that water can form droplets quickly under the interfacial tension between water and lipids will assemble onto the surface of the water droplets to form reverse micelles in the cuboid simulation box (Figure 2 A, see Supporting Information for simulation details). In the second stage, we put the as-fabricated system into the center of a larger cuboid box, and add water beads into it. Similarly, under the interfacial tension between PLGA and water, the PLGA will shrink and form RNV (Figure 2 B). At the same time, several reverse micelles inside PLGA shell can fuse to one bigger micelle, and excess lipids would diffuse to the surface of PLGA shell (Figure 2 C). In the third stage, more lipids added into the system will assemble onto the outer surface of PLGA shell via interaction between PLGA and the hydrophobic tail of lipids.

**Figure 2:**
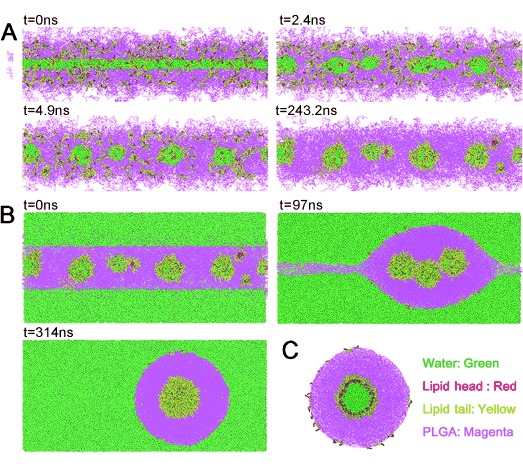
Snapshots of DPD simulation. A) Formation of water droplets, and assembly of lipids onto the surface of water droplets in the first stage of microfluidic chip. Water is shown in green, lipid head in red, lipid tail in yellow, and PLGA in magenta. B) Formation of RNV in the second stage. C) A slice of the formed water core/PLGA shell/lipid layer RNV.

We used a multi-drug resistant cancer model to evaluate the biological efficacy of hydrophilic chemicals delivered by RNV. In this model, the cells express high level of multi-drug resistant (MDR1) protein, which pumps out the anticancer drugs such as doxorubicin (Dox). To knock down MDR1, siMDR1 (the siRNA sequence against the MDR1 mRNA) is utilized in combination with Dox.[14b, 18] With a multi-stage microfluidic chip, we fabricate a co-delivery RNV with siRNA in the water core and Dox in the PLGA shell. The entrapment efficiency of Dox is 96.6±1.1 %, and that of siRNA is 91.23±4.51 % (Table 1, and Supporting Information). The presence of the lipids (DPPC, DSPE-PEG, and cholesterol) as the outermost layer and the high rigidity of RNV may lead to an enhanced cellular uptake.[1] The cellular uptake of RNV is experimentally tested by co-incubation of RNV loaded with siMDR1 and Dox (RNV[Dox/siMDR1]) and MCF-7/ADR cells for 2 h in medium supplemented with 10 % fetal bovine serum (FBS). The measured fluorescence signals from Dox indicate that almost 100 % of MCF-7/ADR cells can uptake RNV[Dox/siRNA], while free Dox can only diffuse into 30 % of cells (Figure 3 A,B, Supporting Information). The ability to carry out the transfection in the presence of serum is a clear advantage for RNV.

**Figure 3:**
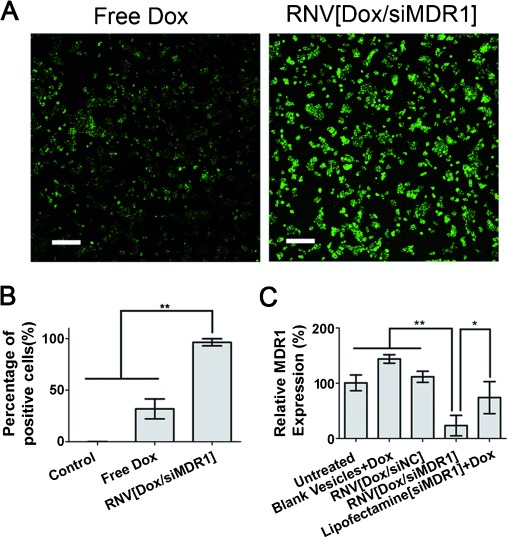
Cellular uptake and in vitro gene silencing effects of RNV. A) Fluorescence images of MCF-7/ADR cells incubated with free Dox and RNV[Dox/siMDR1]. Scale bar, 200 μm. The fluorescence signal is from Dox. B) 2 h cellular uptake of free Dox and RNV[Dox/siMDR1]. C) 72 h gene silencing experiment measured by RT-qPCR. *P<0.05, **P<0.01, siNC: negative control siRNA. Lipofectamine[siMDR1]+Dox: Lipofectamine^2000^ loaded with siMDR1 plus free Dox.

We next evaluate the gene knockdown efficiency by RNV[Dox/siRNA] using the real-time quantitative polymerase chain reaction (RT-qPCR). RNV[Dox/siMDR1] achieves an enhanced gene knockdown efficiency of ca. 80 %, much better than that of the same amount of siMDR1 and Dox delivered by lipofectamine^2000^ (Figure 3 C). In comparison, the blank vesicles plus Dox, and RNV loaded with negative control siRNA and Dox (RNV[Dox/siNC]) display no knock-down effects of MDR1 mRNA. We also investigate the cell apoptosis induced by RNV (Figure S7). The RNV[Dox/siMDR1] achieves the highest anti-tumor efficacy due to an effective suppression of MDR1 gene expression.

In vivo investigation on inhibition of the multi-drug resistant tumor growth is performed on mice via tail vein injection of RNV[Dox/siMDR1], free drugs and other nanocarriers. The blank RNV (BLANK), free Dox plus free siMDR1 (FREE), RNV[Dox/siNC] and PBS control cannot inhibit tumor growth over 13 days. In comparison, a dramatic, consistent suppression of tumor growth is observed when treated with RNV[Dox/siMDR1] at the same dose of siMDR1 and Dox (Figure 4 A–C and Supporting Information). To evaluate the safety of RNV, we measure hemolysis and weight change of mice. The measurement of hemoglobin leaked from red blood cells incubated with RNV[Dox/siMDR1] shows that RNV cannot induce hemolysis (Figure S8). The weight of the mice before and after the treatments kept essentially constant, indicating a low level of toxicity of RNV (Figure 4 D).

**Figure 4:**
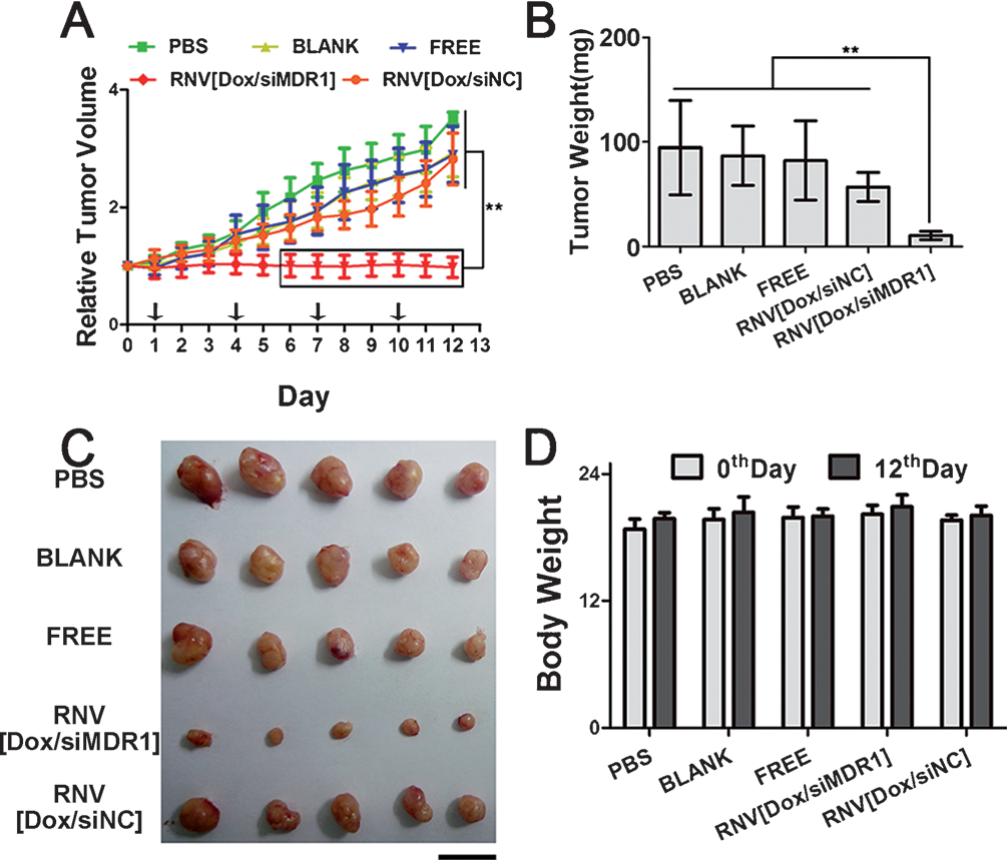
In vivo anti-tumor effects on a multi-drug resistant tumor model. A) Measurement of relative tumor volume growth after treatment. The day before the first dose is specified as day 0. The black arrow on the axis shows the day of administration. B) Weight of the excised tumors. C) Photo of the excised tumors. Scale bar, 1 cm. D) Body weight of the mice before and after the administration. *P<0.05, **P<0.01, siNC: negative control siRNA.

In this work, we develop a three-stage microfluidic platform that can assemble water core/PLGA shell/lipid layer RNV in one step for delivering varying hydrophilic reagents without having to use emulsifiers and stabilizers. For a multi-drug resistant tumor model, an enhanced anti-tumor effect is observed by co-delivering siMDR1 and Dox using RNV in both in vitro and in vivo experiments. RNV also proves to be a safe and biocompatible nanocarrier for in vivo treatment. With these attractive properties, we believe that RNV would be a promising carrier for high efficient delivery of hydrophilic theranostic agents, ranging from cancer treatment to in vivo imaging diagnosis.
