# Supplementary material for: Microfluidic Synthesis of Rigid Nanovesicles for Hydrophilic Reagents Delivery
Source: Angew Chem Int Ed Engl. 2015 Feb 20;54(13):3952–6. doi: 10.1002/anie.201500096 (PMC4471572; doi:10.1002/anie.201500096)
Supplement: Supplementary file 1 [file anie0054-3952-sd1.pdf]

Angewandte Chemie
Eine Zeitschrift der Gesellschaft Deutscher Chemiker

Supporting Information

Microfluidic Synthesis of Rigid Nanovesicles for Hydrophilic Reagents Delivery**

Lu Zhang, Qiang Feng, Jiuling Wang, Jiashu Sun, Xinghua Shi, and Xingyu Jiang**

anie_201500096_sm_miscellaneous_information.pdf

Materials

Poly(D, L-lactide-co-glycolide) (PLGA, lactide:glycolide = 75:25) was purchased from SurModics (USA).1,2-dioleoyl-3-trimethylammonium-propane (DOTAP), cholesterol,1,2-dipalmitoyl-sn-glycero-3-phosphocholine (DPPC) and 1,2-distearoyl-sn-glycero-3-phosphoethanolamine-N-[methoxy(polyethylene glycol)-2000 (DSPE-PEG2000) were purchased from Avanti (USA). Doxorubicin (Dox), rhodamine B, calcein, mannitol, trehalose, agarose, ethidium bromide, ethanol, dimethylsulfoxide (DMSO), dimethylformamide (DMF) and trifluoroethanol (TFE) were purchased from Sigma. siRNAs were synthesized by Ribobio (GuangZhouRibobio CO., LTD) and primers for PCR were synthesized by Life Technologies (Thermo Fisher Scientific) with sequences in Table S1. Hoechst 33342, DiD were purchased from Invitrogen. Roswell Park Memorial Institute (RPMI)1640 medium, fetal bovine serum, penicillin/streptomycin and were purchased from Gibco.MilliQ water (Millipore) was used for all experiments. ReverTra Ace qPCR RT Kit and Thunderbird qPCR Mix were purchased from TOYOBO, Japan. Mouse monoclonal antibody against human of MDR-1 and β-Actin were purchased from Santa Cruz Biotechnology, US.

Microfluidic chip design and fabrication

The chip used to synthesize the RNV has six inlets (100 μm wide×60 μm depth) one outlet (300 μm wide×60 μm depth), two straight mixing channels (300 μm wide×60 μm depth× 5 mm long) and one spiral mixing region (double spiral, 5 loop

each, 300 μm wide×60 μm depth) (Figure S1). SU-8 2050 photoresist (MicroChem Corp) and polydimethylsiloxane (PDMS) (Sylgard 184, Dow Corning Inc.) were used to fabricate the master mold on a silicon wafer. Both the master mold and the chip were fabricated as previously described.[1]

Synthesis of RNV encapsulating hydrophilic molecules

The synthesis initiated from primitive solution introduced by syringe pumps (PHD Ultra, Harvard Apparatus).Hydrophilic molecules, such as siRNA, calcein and rhodamine B, were in aqueous solution with the concentration of 150 μM. In the first stage, this solution was introduced into the chip through the middle inlet at 0.2 mL/hr, and mixed with PLGA and DOTAP (dissolved in dimethylformamide (DMF) and tetrafluoroethylene (TFE) (65/35， v/v) at a mass ratio of 25: 3) introduced from the two side inlets (1 mL/hr each). Two water sheaths were injected from the two side inlets in the second stage (15 mL/hr each), and continued during the whole process. Simultaneously, the lipid solution (DPPC, DSPE-PEG and cholesterol in ethanol with molar ratio of 80: 4: 16) was injected through the middle inlet (0.2 mL/hr) in the third stage. For transmission electron microscopy (TEM) observation, phosphotungstic acid was mixed to the PLGA solution before synthesis at the concentration of 7.5 mg/mL.

Optimization of conditions for synthesis of RNV

According to our preliminary experiments, the flow rate ratio (FR, defined as the flow rates of two side solutions over the flow rate of middle solution) below 10 at the

first stage, or FR below ~15 at the second stage could result in the inefficient mixing inside microchannels, and the precipitated PLGA particles tended to be large in size. We chose FR of 10 at the first stage (side inlets: 1 mL/hr each, middle inlet: 0.2 mL/hr), and FR of ~15 at the second stage (side inlets: 15 mL/hr each) considering the two following factors. (1) The microfluidic chip that we used was made by bonding replica PDMS with the glass substrate. If we further increased the flow rate, the performance of chip might become unstable over a long working period due to the high pressure induced by fluids. (2) If we increased the FR at the first or the second stage, the final concentration of siRNA inside RNV would be decreased, making it less efficient for the following in vitro and in vivo experiments. The production rate of RNV is ~ 114 μg/min (6.832 mg/hr) by the microfluidic chip. For a RNV of a = 140 nm, and ρ =0.5 g/cm^3, the calculated mass is ~ 1×10^{-15} g, and the number of generated particles is ~ 1.14×10^{11} per minute. The amount of RNV generated per hour is enough for 20 doses for one mouse.

Characterization of RNV

The RNV generated with microfluidic chip was characterized with dynamic light scattering(DLS, Zetasizer 3000HS, Malvern Instruments Ltd.) and transmission electron microscopy(TEM, FEI Tecnai T20). The hydrodynamic diameter and zeta potential of particles were measured by DLS at a scattering angle of 173°. The structure of RNV was observed with TEM (acceleration voltage 200 kV) by dropping RNV suspension onto a carbon film-coated grid and then air-dried at room

temperature before imaging.

Characterization on encapsulation of hydrophilic argents by RNV

The fluorescence spectrum of RNV encapsulating calcein and rhodamine B was obtained with spectrofluorophotometer (RF-5301PC, SHIMADZU) with excitation at 455 nm for calcein and 556 nm for rhodamine B. The spectrum of free dyes was recorded at the same wavelength and compared with that of RNV. The results indicated that the dyes were successfully entrapped into the RNV, because the fluorescence spectrum of RNV was the same as that of free dyes (Figures 1B, 1C, S2, and S3).

Characterization on encapsulation efficiency

For determining encapsulation efficiency, the RNV entrapping hydrophilic molecules (calcein and rhodamine B) was filtered with Amicon Ultra-0.5ultrafilter (MWCO=30KDa, Millipore, USA). The concentration of free calcein or rhodamine B in the diffusate was measured by detecting the fluorescence intensity at 518 nm (excitation wavelength 488 nm for calcein) or 590 nm (excitation wavelength 548 nm for rhodamine B) with infinite M200 microplate reader. The encapsulation efficiency of dyes equals to $(Q_{total}-Q_{free})/Q_{total}$, in which Q_{total} is the total amount of dyes and Q_{free} is the amount of free dyes in diffusate. The amount of siRNA encapsulated in the RNV and free siRNA were determined by agarose gel electrophoresis assay. The agarose gel after electrophoresis was stained with ethidium bromide and imaged with

Image Quant 3000. The image was analyzed with ImageJ 2x (NIH) to quantify the fluorescence of siRNA bands.

Lyophilization of RNV

For long-term storage, the RNV was suspended in aqueous solution with 1% mannitol and 10% trehalose and lyophilized in vacuum at -60 ^{o}C for 24 h with a FD-1A-50 vacuum freeze dryer (Boyikang, Beijing) to remove water and DMF.

There is almost no change of RNV size and polydispersity index (PDI) after lyophilization (Table S2). TEM images of RNV[Dox/siMDR1] before and after lyophilization also verify that lyophilization will not affect the complex structure of RNV (Figure S4).

***In vitro* release of Dox**

In vitro release of Dox from the RNV[Dox/siMDR] was performed at pH 7.4 and pH 4.5 in phosphate buffer saline (PBS) at 37 ^{o}C. In brief, 150 μL of RNV[Dox/siMDR] or Dox in DMF solution (final concentration of Dox was 50 μg/mL) was placed in a dialysis bag (cut-off mass 12,000 -14,000). The dialysis bags were maintained in 3 mL PBS and shaken (100 rpm) at 37 ^{o}C. At determined time point, we replaced the medium with 3mL fresh PBS, and collected the old medium containing released Dox. After 72 hr, the samples in dialysis bags were completely mixed with the medium for the measuring of total Dox concentration. All samples were lyophilized and dissolved with DMF. The concentration of Dox in different

samples was determined by fluorescent intensity (excitation at 490 nm, emission at 590 nm) by an Infinite M200 microplate reader (Tecan). The cumulative release rate at each time point was calculated according to the formula: release rate at time i (%) = (sum of Dox concentration before i)/ (sum of Dox concentration at 72hr) × 100%. Under the condition of pH 7.4, the release rate of Dox encapsulated by PLGA shell was relatively slow and the total amount of released Dox was below 40 % even after 72 hr (Figure S5). In comparison, the release rate of encapsulated Dox was faster at pH 4.5. After 48 hr, more than 60 % of Dox was released (Figure S5). The intracellular pH of tumor cells, especially in lysosomes, is generally below 5, so we speculate that 72 hr is sufficient for the degradation of PLGA. However, we found that it is hard to measure the release curve of siRNA inside the RNV because the siRNA tends to quickly degrade after releasing to the environment. Based on the *in vitro* gene silencing experiment (Figure 3C) and the degradation of PLGA, we believed that the release of siRNA occurred within 72 hr.

Dissipative particle dynamics (DPD) simulations

Because the intermediate steps of the formation of the particles and the synthesis process of RNV within the microfluidic channel are difficult to capture and characterize using experimental approaches, we carryout dissipative particle dynamics (DPD) simulations.[2] It is shown that DPD is a very useful method to study biological systems, especially for biomembrane systems.[3] In the DPD simulation, the force on bead *i* due to bead *j* is given as a sum of 3 terms:

$$F_i = F_{ij}^C + F_{ij}^D + F_{ij}^R, r_{ij} < r_c \qquad (1)$$

where F_{ij}^{C} is a conservative force, F_{ij}^{D} is a dissipative force, F_{ij}^{R} is a random force, r_{ij} is the distance between beads *i* and *j*, and r_c is the cutoff distance. (If r_{ij} exceeds r_c, there would be no interaction between *i* and *j*.). The conservative force acts to give beads a chemical identity, while the dissipative and random forces together form a thermostat that keeps the mean temperature of the system constant. The conservative force between beads *i* and *j* is soft repulsive and determined by:

$$F_{ij}^{C} = a_{ij}\hat{r}_{ij} max\left\{1 - {}^{r_{ij}}/_{r_c}, 0\right\} \quad (2)$$

where a_{ij} is the maximum repulsive strength, $\hat{r}_{ij} = \boldsymbol{r}_{ij}/r_{ij}$ is the unit vector.The interaction parameters are shown in Table S3.

We adopt the lipid model developed by Groot and Rabone.[4] In this model, the lipids molecule is represented by the $H_3(T_5)_2$ model, where the head of lipid molecule is formed by three hydrophilic beads (H), and the tails is formed by five hydrophobic beads (T). The neighboring beads *i* and *j* in the lipid are connected together by a simple harmonic spring:

$$U_s = K_s(1 - r_{ij}/r_s)^2 \quad (3)$$

where spring constant $K_s = 50k_BT$, and equilibrium bond length $r_s = 0.7r_c$. We also apply a harmonic constraint on the adjacent three beads, and details can be seen in the paper by Li *et al*.[5] Comparing to a typical membrane thickness of 4 nm, the basic length unit, r_c, in the simulation is about 0.8 nm. By mapping the diffusion coefficient around for 5 μm^2/s for lipids,[6] the time unit $\tau = [m{r_c}^2/(k_BT)]^{1/2}$, is about 24.32 ps.

We use 200 connected beads for PLGA chains. The neighboring beads in PLGA are also connected together by a harmonic spring with spring constant $K_s = 200k_BT$, and equilibrium bond length $r_s = 1.0r_c$.

Step1: generation of water droplets in PLGA

Firstly, we study the process to mix water, lipids, PLGA. The simulation box is a cuboid of size $20r_c$×$20r_c$× $110r_c$, with periodic boundary condition applied along *x*, *y* and *z* directions. The system consists of 400 lipid molecules, 613 PLGA chains and 4200 water beads, as shown in Table S4. There are totally 132,000 beads in the system with particle density about 3.0. We put a relaxed water bead into a cylindrical column with a diameter of $4r_c$ in the center of the box and other beads outside the water column. At the beginning of the simulation, we fix the water and let other beads relax. (At this step, to ensure lipids randomly distributed in PLGA, we have set a_{ij} between water and other beads as 100, and a_{ij} among other beads except for water as 25.) Then we release the water and perform DPD simulations. We can see water form droplets quickly under the interfacial tension between water and PLGA (Figure 2). Afterwards, lipids will assemble at the surface of the water droplets, and form reverse micelles.

Step2:Generation of RNV

Then we study the process when we add more water to the system. We construct a larger system with box size $50r_c$× $50r_c$× $110r_c$, and put the as-fabricated system in the first step to the center of the box. We add water bead with beads density of 3.0 to the system. At this moment, the system consists of 825,000 beads in total. Periodic boundary condition is also applied along *x*, *y* and *z* directions. Figure 2 shows snapshots of the simulation process. Similarly, under the interfacial tension between PLGA and water, the PLGA will shrink and form RNV. Since the chains of PLGA is long (200 beads in one chain), we can see the RNV will extract the PLGA chains from each other. At the same time, the reverse micelles would fuse to one bigger micelle, and excess lipid would diffuse to the inner surface of the PLGA shell. In the

third stage, more lipids added into the system will assemble onto the outer surface of PLGA shell to form the water core/PLGA shell/lipid layer RNV.

Surface charge of RNV

Two kinds of lipids are involved for the generation of RNV. One is the cationic lipid (DOTAP, with positive surface charge) that is introduced into the chip from the inlet of first stage and entrapped by the PLGA shell. The other is the neutral lipid (DPPC, cholesterol and DSPE-PEG, with negative surface charge), which is injected from the inlet of the third stage and assembles onto the outer surface of the PLGA shell. DPPC, cholesterol and DSPE-PEG are generally divided to the category of neutral lipids. However, after the forming of liposomes, especially under the environment of pH 7.4, the nanoparticle shows a slightly negative surface charge as the result of the PEG chain. This phenomenon was widely reported in many papers.[7] The excess cationic lipids due to the fusion of reverse micelles may diffuse to the outer neutral lipid layer, resulting in the positive surface charge of RNV when encapsulating calcein and rhodamine B. However, for fabrication of RNV encapsulating siRNA, the negatively charged siRNA will interact with the positively charged DOTAP, thus limiting the diffusion of DOTAP into outer lipid layer and resulting in the negative surface charge of RNV.

Cell Culture

Dox-resistant human breast cancer cells MCF-7/ADR were obtained from the

Institute of Hematology & Blood Diseases Hospital (Tianjin, China). The cells were maintained in RPMI-1640 medium (Gibco, NY, USA) supplemented with 10% fetal bovine serum (Gibco, NY, USA), 100 units/mL penicillin and 100μg/mL streptomycin at37 ^{o}C in humidified atmosphere containing 5% CO_2.

Animals

Female BALB/c nude mice (18-20 g) were purchased from Vital River Laboratory Animal Center (Beijing, China). All care and handling of animals were performed with the approval of Institutional Authority for Laboratory Animal Care of Institute of Process Engineering, Chinese Academy of Science. The animals were raised in a specific pathogen free (SPF) environment.

Characterization of drug resistant cells

The cytotoxicity of Dox to MCF-7 and MCF-7/ADR cells was measured to evaluate the Dox sensitivity in MCF-7/ADR cells. Briefly, MCF-7 cells and MCF-7/ADR cells were seeded into 96-well plates at a density of 4×10^3 per well, incubated for 24hr before use. The medium was replaced with fresh RPMI-1640 with different concentration of Dox. Cell viability was measured at 48 hr via the SRB assay,[8] and the absorbance was read on a microplate reader at 540 nm. The cell viability (%) was calculated according to the following formula: Cell viability (%) = [OD540 (sample)/OD540 (control)] × 100, where OD540 (sample) is the absorption from the wells treated with samples and OD540 (control) is that from the wells treated

with fresh medium with PBS.

To further evaluate the expression of MDR1 protein, different cells were lysed in radio immunoprecipitation assay (RIPA) lysis buffer with 10 mM PMSF. Western blot assay was performed to determine the MDR1 protein in cancer cells. The proteins were resolved by the SDS-PAGE, transferred to the PVDF membrane, blocked in 5% skim milk in TBST, and blotted with the antibodies for MDR1 (1:250) and β-actin (1:250) (Figure S6).

Cellular uptake

Flow cytometry analysis (FACScan flow cytometer, Beckman Quanta SC, US) was used to provide quantitative results of cellular uptake of the RNV. MCF-7/ADR cells were seeded into 6-well plates at a density of 3×10^5 cells per well, and cultured in RPMI 1640 medium supplemented with 10% FBS. After 24h incubation, the cells were washed with phosphate-buffered saline (PBS) twice and Dox labeled RNV in freshRPMI 1640 medium supplemented with 10% FBS were separately added to each well for transfection, with a final siRNA concentration of 100nM. After incubation for 2hr, the cells were washed with PBS and detached with trypsin. Then, cells were washed by PBS for three times and the fluorescence intensity of Dox was measured using flow cytometry scanner (FCS). Cells treated with PBS were used as negative control.

Gene silencing efficiency

MCF-7/ADR cells were seeded into 6-well plates at a density of 2×10^5 cells per flask, incubated for 24hr before use. The wells were washed with PBS twice and different RNV containing 100nM siMDR1and 5 μg/mL Dox in 2mL fresh RPMI 1640 premium supplemented with 10% FBS were separately added to each well for transfection. The incubation was continued for 60hr and the cells were washed 3 times with pre-chilled PBS.

Total RNA was extracted using TRIzol® reagent method and reverse transcribed with ReverTra Ace qPCR RT Kit. The resulting cDNAs were used for qPCR using Thunderbird qPCR Mix in triplicates. qPCR and data collection were performed on Real time PCR amplifier (Eppendorf). All quantitation were normalized to an endogenous control β-Actin. The relative quantitation value for each target gene compared to the calibrator for that target is expressed as $2^{-(\Delta C_t-\Delta C_c)}$($\Delta C_t$ andΔC_c are the mean threshold cycle differences after normalizing toβ-Actin). The experiments were carried out for 3 times and the results were shown as mean ± SD.

Cell apoptosis

Cell apoptosis was quantified by Annexin V-FITC/PI assay. MCF-7/ADR cells were seeded in 6-well plates and treated with different nanoparticles or free drug in complete medium. The final concentration of siRNA is 100nM while that for Dox is 5 μg/mL. After 72hr incubation, all cells were harvested and stained with Annexin V-FITC and PI following the manufacturer's instructions. Fluorescence of cells was measured using a FACScan flow cytometer (Beckman Quanta SC, US).

Hemolysis assay

The 2% (v/v) red blood cells (RBC) solution was obtained by re-suspending the cell pellet to pre-chilled PBS solution and then seeded (160μL) into 96-well plates. 40 μL of the RNV[Dox/siMDR1] solution and lipofectamine^{2000}/siRNA solution (with the same siRNA concentration of 100nM) were added to each well and the mixture was incubated for 1hr at 37 ^{o}C. After that, intact RBCs were removed by centrifugation and the hemoglobin released into supernatant was measured by absorbance at 540 nm as an indication of membrane disruption. Triton X-100 (1%, v/v) and PBS were used as positive and negative controls, respectively.

In vivo tumor growth inhibition study

Female Balb/c mice (6–8 weeks old), weighing 18–22 g were purchased from Vital River Laboratory Animal Center (Beijing, China). 5×10^6 MCF-7/ADR cells were inoculated subcutaneously(s.c.) in the right flank of the Balb/c nude mice[9]. When tumor size reached 120–150 mm^3 in volume, animals were sacrificed and tumors were aseptically dissected and minced with scissors into 15mm^3 pieces. Then, tumor tissues were transplanted s.c. into the armpit of the mice. When the tumor volume was above 70 mm^3, mice were randomly divided into 5 groups (5 animals per group): (1) PBS, (2)BLANK (RNV[H_2O]), (3) FREE (PBS+ FreeDox +Free siMDR1), (4) RNV[Dox/siMDR1], (5) RNV[Dox/siNC]. The dose of Dox was 4 mg/kg and that of siRNA was 1 mg/kg. Each dose was given every three days (total for 4 doses) by tail

vein injection. The day before the first dose was specified as day 0. Tumor size was measured daily using a vernier caliper across its longest (L) and shortest (S) diameters and the volume was calculated according to the formula of $V = 0.5LS^2$. Body weight of the animals was measured at day 0 and day 12. Two days after the last injection, the animals were sacrificed, and the tumor tissues were weighed.

We have not included the control of RNV[siMDR1] (RNV has the active siRNA against MDR1 but lacks Dox) because the previous investigation showed that nanoparticles encapsulating siMDR1 had no anticancer effects.[10] siRNA against MDR1 is not expected to have a therapeutic effect in the absence of a chemotherapeutic to take advantage of the change in protein translation with siRNA knockdown.

There might be two main reasons for the fact that RNV[Dox/siMDR1] did not eradicate the tumor, but rather just prohibited its growth. (1) The dead tumor cells still occupied some space in the tumor site, making the tumor have the similar size as before treatment. [11] (2) RNV[Dox/siMDR1] might not penetrate or spread through the whole tumor tissue so there was a possibility that some parts of tumors were still growing. This matches the result of some previous work. We did not monitor the animals longer because we observed a significant difference between the RNV[Dox/siMDR1] and other groups at day 10, so we terminated the experiments only two days after the last dose mainly considering the animal welfare in order to reduce the time of animals suffering from cancer.[12]

Statistical analysis

For statistical analysis between two groups, Student's t-test for independent means was applied. The differences between any two groups out of several groups were analyzed by one-way analysis of variance (ANOVA) followed by Tukey multiple comparisons. Statistical analysis was performed with the SPSS software (Version 16.0, SPSS Inc, Chicago). A value of $P < 0.05$ was considered as statistically significant.

References

[1] J. Sun, L. Zhang, J. Wang, Q. Feng, D. Liu, Q. Yin, D. Xu, Y. Wei, B. Ding, X. Shi, X. Y. Jiang, *Adv. Mater.***2014**, DOI:10.1002/adma.201404788.

[2] R. D. Groot, P. B. Warren, *J. Chem. Phys.* **1997**, *107*, 4423-4435.

[3] a) K. Yang, Y. Q. Ma, *Nat. Nanotechnol.* **2010**, *5*, 579-583;b) Y. F. Li, H. Y. Yuan, A. von dem Bussche, M. Creighton, R. H. Hurt, A. B. Kane, H. J. Gao, *Proc. Natl. Acad. Sci. U. S. A.* **2013**, *110*, 12295-12300;c) T. T. Yue, X. R. Zhang, *ACS Nano* **2012**, *6*, 3196-3205.

[4] R. D. Groot, K. L. Rabone, *Biophys. J.* **2001**, *81*, 725-736.

[5] Y. Li, M. Kroger, W. K. Liu, *Biomaterials* **2014**, *35*, 8467-8478.

[6] J. C. Shillcock, R. Lipowsky, *Nat. Mater.* **2005**, *4*, 225-228.

[7] a) A. L. Bailey, S. M. Sullivan, *Biochimica et Biophysica Acta* **2000**, 1468, 239-252; b) W. Zhao, S. Zhuang, X. R. Qi, *Int. J. Nanomed.* **2011**, 6, 3087-3098.

[8] V. Vichai, K. Kirtikara, *Nat. Protoc.***2006**, *1*, 1112-1116.

[9] P. A. Foster, S. P. Newman, S. K. Chander, C. Stengel, R. Jhalli, L. L. Woo, B. V. Potter, M. J. Reed, A. Purohit, *Clin. Cancer. Res.***2006**, *12*, 5543-5549.

[10] J. A. Ludwig, G. Szakacs, S. E. Martin, B. F. Chu, C. Cardarelli, Z. E. Sauna, N. J. Caplen, H. M. Fales, S. V. Ambudkar, J. N. Weinstein, M. M. Gottesman, *Cancer Res.* **2006**, 66, 4808-4815.

[11] J. Jiang, S. J. Yang, J. C. Wang, L. J. Yang, Z. Z. Xu, T. Yang, X. Y. Liu, Q. Zhang, *Eur. J. Pharm. Biopharm.* **2010**, 76, 170-178.

[12] D. J. Mellor, K. J. Stafford, *Aust. Vet. J.* **2001**, *79*, 762-768.

[13] V. Stierle, A. Laigle, B. Jolles, *Biochem. Pharmacol.***2005**, *70*, 1424-1430.

[14] H. Meng, W. X. Mai, H. Zhang, M. Xue, T. Xia, S. Lin, X. Wang, Y. Zhao, Z. Ji, J. I. Zink, A. E. Nel, *ACS Nano***2013**, *7,* 994-1005.

Table S1.Sequencesof siRNAsand primers

Name	Sequence (5'-3')
siMDR1(sense strand)[13]	GAA ACC AAC UGU CAG UGU AdTdT
siNC(sense strand)	UUCUCCGAACGU GUCACGUdTdT
Forward primer β-Actin[13]	ACC AAC TGG GAC GAC ATG GA
Reverse primer β-Actin[13]	CTC CTT AAT GTC ACG CAC GCA CGA
Forward primer MDR1[14]	AGG AAG CCA ATG CCT ATG ACT TTA
Reverse primer MDR1[14]	CAA CTG GGC CCC TCT CTC TC

Table S2. Lyophilization of RNV

	Size (d,nm)	PDI
Before lyophilization	200.9±3.4	0.178±0.014
After lyophilization	197.8±2.4	0.203±0.023

Table S3. The interaction parameters a_{ij} between different type of beads

a_{ij}	Lipids head	Lipids tail	PLGA	Water
Lipids head	25	100	100	25
Lipids tail	100	25	25	100
PLGA	100	25	25	100
Water	25	100	100	25

Table S4. The simulation system

	molecular numbers	atom numbers in one molecular
Lipids	400	13
PLGA	613	200
Water	4200	1

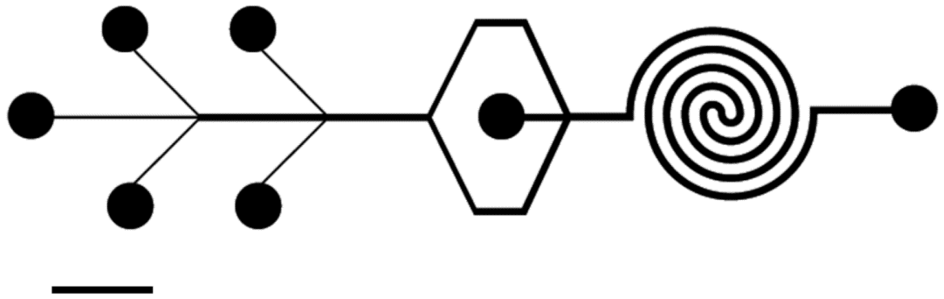

Figure S1. CAD draw of the multi-stage microfluidic chip for generating RNV.Scale bar, 5 mm.

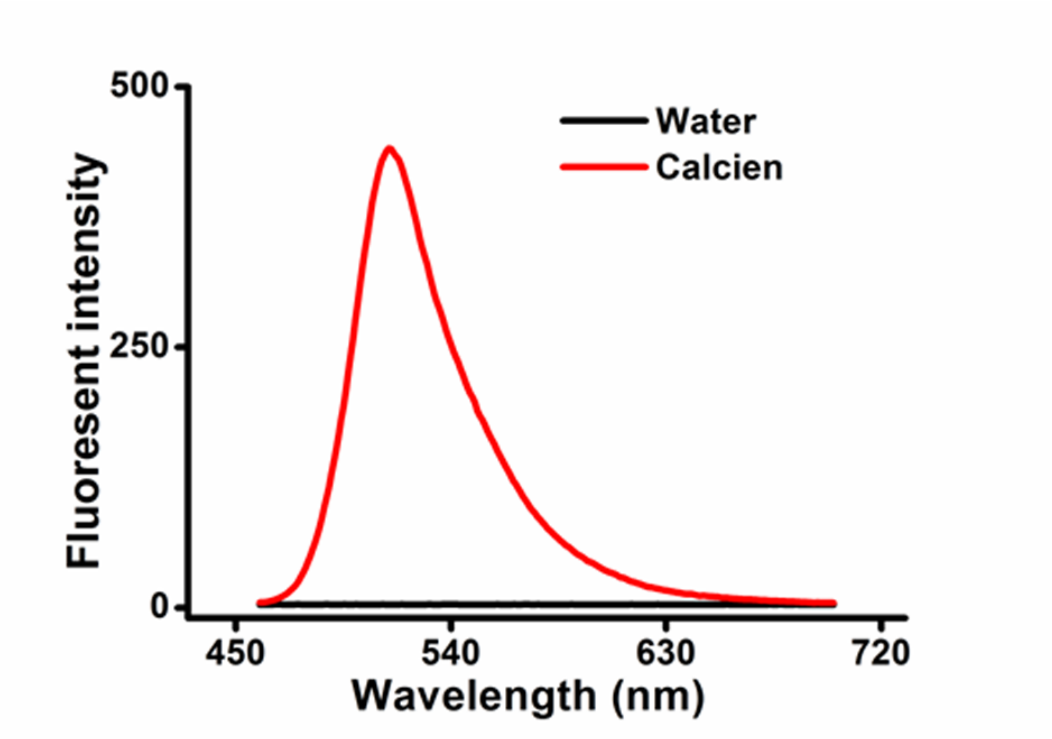

Figure S2. Fluorescence spectrum of calcein in aqueous solution. Excitation wavelength is 455 nm.

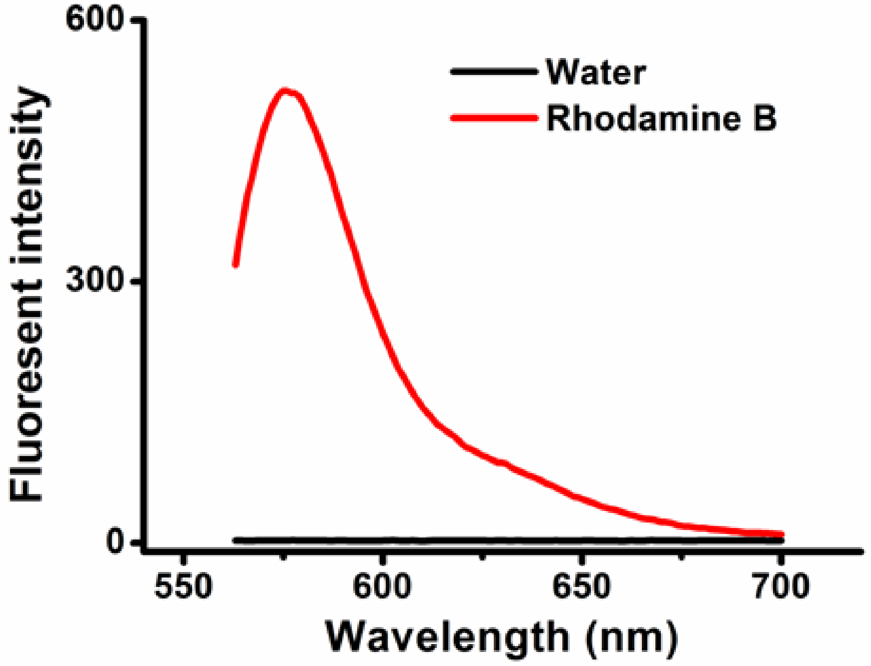

Figure S3. Fluorescence spectrum of rhodamine B in aqueous solution. Excitation wavelength is 556 nm.

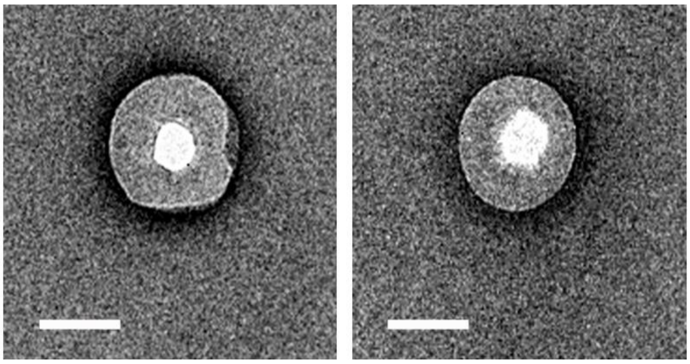

Figure S4. TEM images of RNV[Dox/siMDR1] before (left) and after (right) lyophilization. Scale bar, 100 nm.

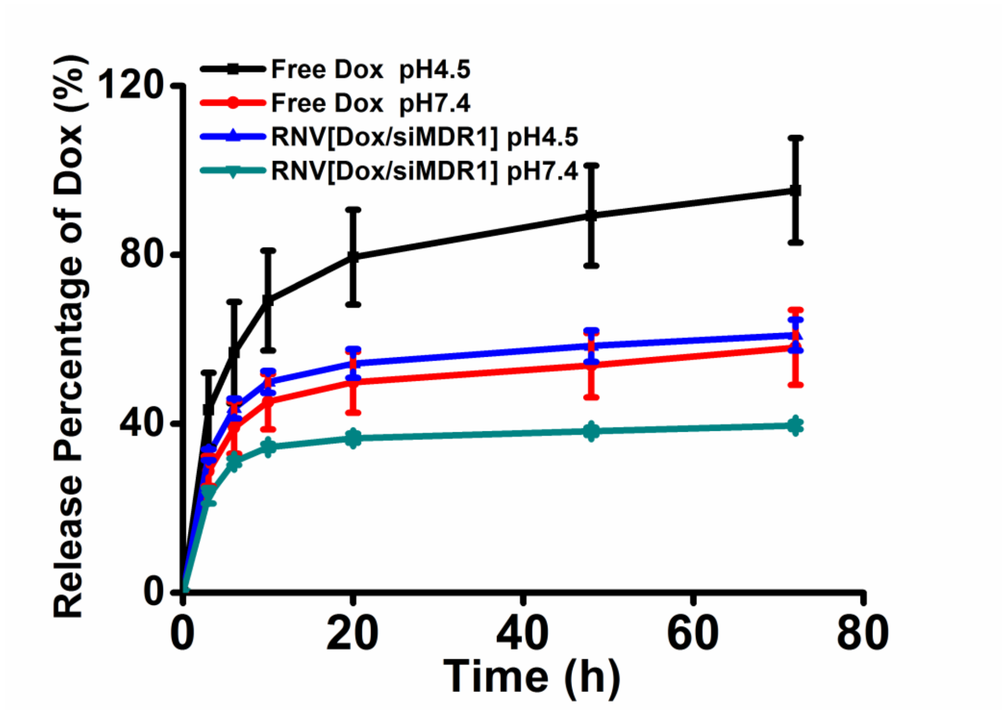

Figure S5. Evaluation of the release rate of Dox in the PLGA shell of RNV[Dox/siMDR1] under different pH conditions. Free Dox is used as control. See SI text (pg. S-6) for details.

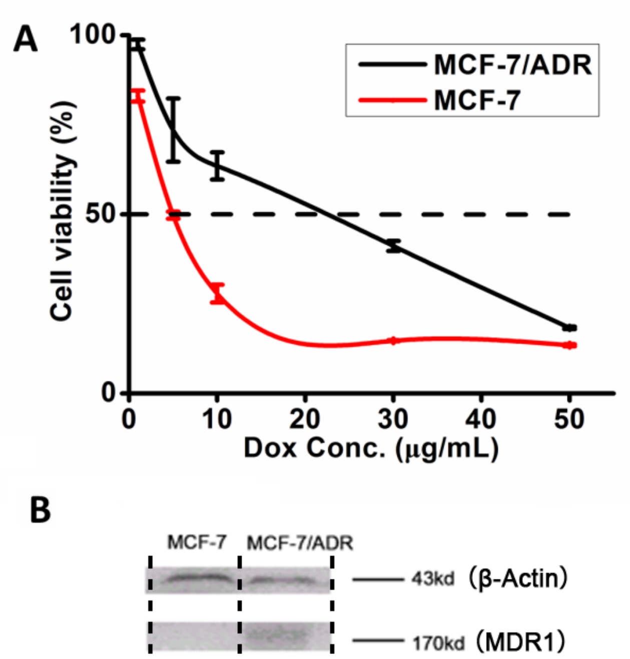

Figure S6. Characterization of drug resistant cells. A) The cytotoxicity of Dox to MCF-7 and MCF-7/ADR cells. B) Western graph of the MDR1 proteinin MCF-7 and MCF-7/ADR cells. The protein ladder was not shown because it was cut off after the proteins were transferred to the PVDF membrane. Since the blotting was conducted using the antibodies of Actin and MDR1 respectively, the bands could only be observed if we used the correct area of PVDF membrane.

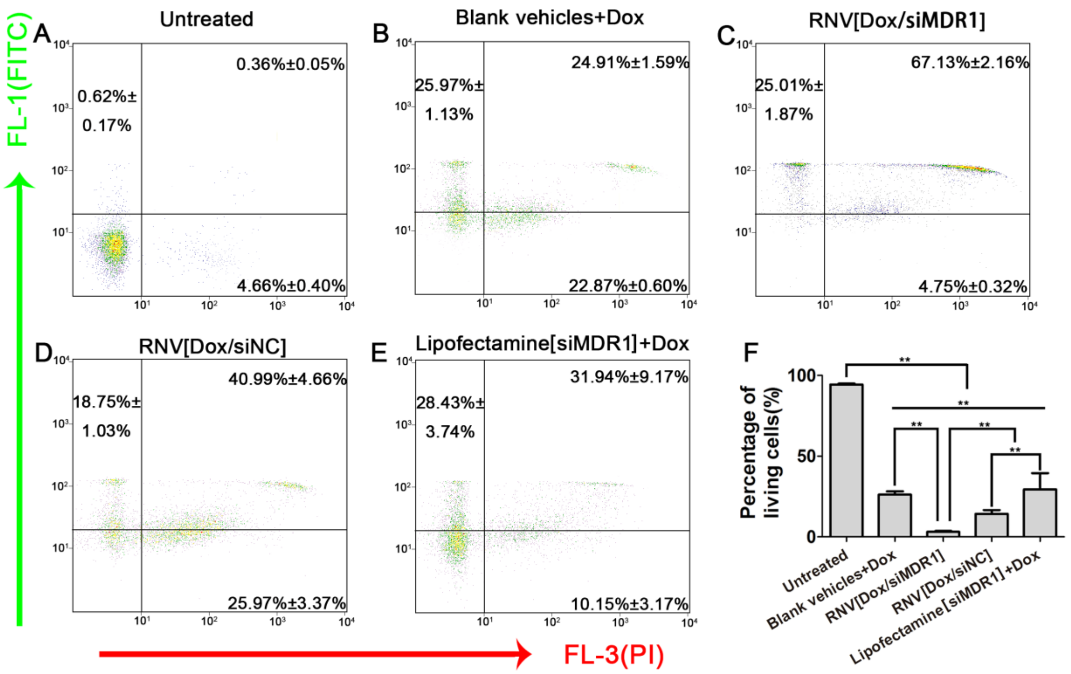

Figure S7. Cell apoptosis of MCF-7/ADR cells induced by (A) None, (B) RNV[H_2O] plus free Dox, (C) RNV[Dox/siMDR1], (D) RNV[Dox/siNC], (E) Lipofectamine[siMDR1]+Dox. F) The percentage of living cells calculated from figure A-E.The vertical axis is the fluorescence of Annexin V-FITC (Ex 488 nm, Em 525 nm), indicating the amount of phosphatidylserineon the outside of cell plasma membrane. The horizontal axis is the fluorescence of propidiumiodide (PI, Ex 488 nm, Em 575 nm)), indicating the cell membrane permeability. The cells with no fluorescence of Annexin V-FITC and PI (Annexin V-/PI-) are living cells.The cells with Annexin V+/PI-areviable apoptotic cells， while those with Annexin V+/PI+ and Annexin V-/PI+ arenon-viable apoptotic cells and necrotic cells respectively. These data shown that RNV[Dox/siMDR1] induced apoptosis of MCF-7/ADR cells and the percentage of living cells in this group is significantly lower than that in the other groups.

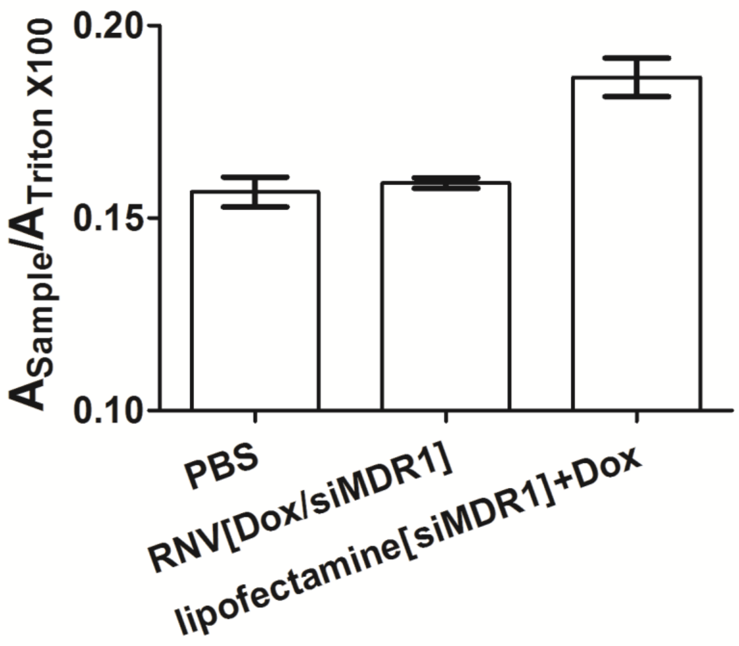

Figure S8. Hemolysis assay based on 2% (v/v) RBC solution. The absorbance at 540 nm indicates the amount of the hemoglobin released into supernatant. Triton X-100 (1%, v/v) is used as positive control. The similar value compared to PBS group reveals that RNV[Dox/siMDR1] induces no hemolysis effect.

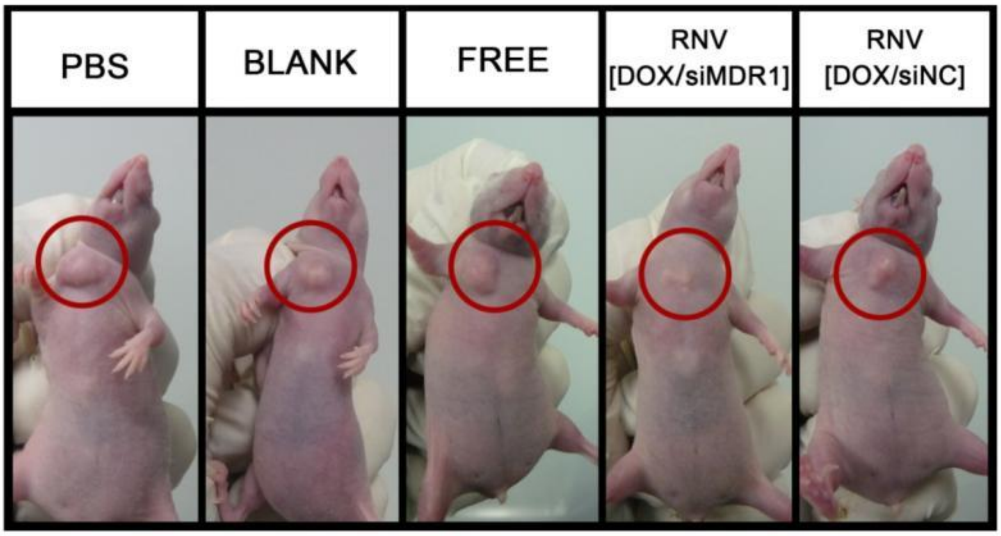

Figure S9. The photographs of Balb/c mice after 4 doses tail vein injection. Red circle marks the position of tumors.
